# Community-based physical activity interventions among women: a systematic review

**DOI:** 10.1136/bmjopen-2014-007210

**Published:** 2015-04-01

**Authors:** Leila Amiri Farahani, Mohsen Asadi-Lari, Eesa Mohammadi, Soroor Parvizy, Ali Akbar Haghdoost, Ziba Taghizadeh

**Affiliations:** 1 Department of Midwifery, School of Nursing and Midwifery, Tehran University of Medical Sciences, Tehran, Iran; 2 DG Int'l Relations Department, MOHME, IR Iran Head- Oncopathology Research Centre, IUMS, Tehran, Iran; 3 Nursing Department, Faculty of Medical Sciences, Tarbiat Modares University, Tehran, Iran; 4 Department of Pediatric Nursing, Nursing and Midwifery Faculty, Iran University of Medical Sciences, Tehran, Iran; 5 Centre for educational Research in Medical Sciences (CERMS) Iran University of Medical Sciences, Tehran, Iran; 6 The Research Centre for Modelling in Health, Institute for Future Studies in Health, Kerman University of Medical Sciences, Kerman, Iran; 7 Faculty Member of Nursing and Midwifery Care Research Centre, Nursing and Midwifery School, Tehran University of Medical Sciences, Tehran, Iran

## Abstract

**Objective:**

Review and assess the effectiveness of community-based physical activity interventions among women aged 18–65 years.

**Design:**

Systematic review

**Methods:**

To find relevant articles, the researcher selected reports published in English between 1 January 2000 and 31 March 2013. Systematic search was to find controlled-trial studies that were conducted to uncover the effect of community-based interventions to promote physical activity among women 18–65 years of age, in which physical activity was reported as one of the measured outcomes. The methodological quality assessment was performed using a critical appraisal sheet. Also, the levels of evidence were assessed for the types of interventions.

**Results:**

The literature search identified nine articles. Four of the studies were randomised and the others studies had high methodological quality. There was no evidence, on the basis of effectiveness, for social cognitive theory-based interventions and inconclusive evidence of effectiveness for the rest of interventions.

**Conclusions:**

There is insufficient evidence to assess the effectiveness of community-based interventions for enhancing physical activity among women. There is a need for high-quality randomised clinical trials with adequate statistical power to determine whether multicomponent and community-based intervention programmes increase physical activity among women, as well as to determine what type of interventions have a more effective and sustainable impact on women's physical activity.

**Strengths and limitations of this study**

- This is the first review to explore the effectiveness of community-based physical activity interventions among women aged 18–65 years.
- The trial screening and data extraction were conducted using the strong appraisal sheets, independently by two authors.
- Owing to heterogeneity in the types of community-based interventions, methodology quality and the impossibility of searching all electronic and non-electronic databases with a language restriction, the ability of achieving strong (solid) conclusions might be limited.

## Introduction

Physical activity (PA) is recognised as one of the most important behaviours for reducing the overall burden of disease in humans.^1^ The leading causes of death worldwide are primarily found among four non-communicable diseases (NCDs): cardiovascular diseases, cancers, diabetes and chronic respiratory diseases. The burdens of these diseases are considerably heavier in developing and low-income countries where the rates of these NCDs continue to climb.^2^ Developing countries have been experiencing a rapid phase of unplanned urbanisation and industrialisation, population-ageing and globalisation. These result in unhealthy environments, with rapid social and economic transition accompanied by changes in PA. As a result, the growing prevalence of NCDs and their risk factors has become a global issue in undeveloped and developing countries.^3^

By 2030, low-income countries will have eight times more deaths attributed to NCDs than high-income countries.^2^ The WHO estimates that 80% of all deaths may be attributed to NCDs by 2020.^4^

Tobacco use, harmful use of alcohol, insufficient PA and unhealthy diet are the four main behavioural risk factors which induce NCDs and are expected to rise in developing countries.^5^

In reference to the US physical activity guideline (2008), there is strong evidence that PA reduces the risk of many adverse health outcomes, such as early death, coronary heart disease, stroke, high-blood pressure, adverse blood lipid profile, type 2 diabetes, metabolic syndrome and depression;^1^ also PA is considered an independent cancer-protective factor.^6^

Although there are many benefits in adopting PA, its rates have remained low.^7^ Dumith *et al*^8^ have presented a comprehensive worldwide estimation of physical inactivity in which the overall prevalence of physical inactivity was 21.4%, that is, 23.7% prevalence for women and 18.9% prevalence for men; however, their report was limited by many factors, such as not having access to data from many populous countries, and using a self-report questionnaire that caused underestimation of physical inactivity.

According to the second report of the urban health equity assessment and response tool (Urban HEART) project conducted in 2011 in Tehran, Iran, only 20.5% women and 24.3% men exercise at least for the minimum time recommended by the guideline of PA(unpublished data). In this guideline, 150 min of moderate intensity exercise or 75 min of vigorous intensity exercise is considered as the minimum PA per week.^1^

The lower PA rate among women can be explained by gender-norm limitations that they face in their life. The limitation includes child care responsibility, security, lack of time, lack of confidence on their physical abilities, lack of knowledge about designing and maintaining a PA programme, traffic restrictions, financial inability,^9^ traditional views about women, weather condition, uncomfortable workout cloths and individual motivation.^10^

Iranian women encounter exceptional social and cultural constraints, such as disagreement with their spouse or father about going to gyms or their participation in PA. There are also some sociocultural expectations, and environmental and religious constraints, such as banning females from biking or exercising outdoor.^11^ As they play an important role in the nurturing and upbringing of children, being physically active is very important for women's health and could help to have healthy future generations. Undoubtedly, lack of PA among females can cause unrecoverable damages to the society as it negatively affects physical and mental health of women, a half of the population. This shows the necessity of improving PA in women.^12^

Although the benefits of PA are now well-established, there is not much established knowledge regarding the effectiveness of interventions designed to improve population PA.^13^

The fast growth of chronic diseases in developing countries has increased the awareness of correcting lifestyle inactivity and encouraged community-based interventions. Community-based interventions provide a cost-effective and reasonable way to promote health and access to PA resources for large groups of people, especially when there are limited resources within the community.^14–16^

Community-based interventions are multilevel approaches and use an ecological perspective. Such interventions can be implemented at any of the four ecological levels: group, organisation, community and policy. Three theories that have been used frequently in community-based approaches are the social cognitive theory (SCT), stages of change theory and social marketing theory.^17^

According to Bopp and Fallon,^14^ community-based PA interventions involve community members and leaders from various settings and organisations (ie, at any of the four ecological levels) in the design, implementation and evaluation of a PA intervention. Owing to community members’ involvement in the plan, implementation and evaluation of community-based interventions, these interventions can be more effective and sustainable than individual interventions.^18^ ^19^

The majority of interventions have been delivered at the individual level to change only the personal behaviour.^20^ Although some individual-level and face-to-face interventions are effective as well as the gold standard for promoting PA, transferring and delivering individual-level interventions to community-level is challenging.^21^ It is necessary to run the community-level interventions, which have the potential to produce long-term benefits, for a large number of people, but there is no strong evidence which type of community-based interventions are most effective.^22^

Although many interventions to improve PA are being carried out with women between 18 and 65 years of age, the types and effectiveness of most interventions have not been systematically examined. Recently published reviews have mostly dealt with the increase PA among both genders or only included underserved and/or minority women. Previous endeavours to summarise the evidence were mostly allocated to particular settings and individual interventions. They also did not assess the effects of interventions on women with a community-based approach and did not assess the methodological quality of the studies.

This paper describes a systematic literature review of strategies for promoting PA among women aged 18–65 years, and conducted with community-based approaches. This review is a small part of a larger project entitled Improving PA among Women: a Mixed-method Action Research in Iran. The overarching goal of this project is to develop a community-based interventions programme for promoting PA among women in Iran.

## Methods

### Search strategy and inclusion criteria

To the best knowledge of the author of this article, all documents, including articles, theses and conference abstracts, that were published between 1 January 2000 and 31 March 2013 in electronic databases, such as PubMed, Science Direct, Google scholar and Cochrane Library were searched.

The search strategy was created and run by LAF with assistance from the library and an information science expert. Keywords and combinations (MeSH and text words), such as physical activity, physical inactivity, exertion, fitness and community-based intervention, community-based research and population-based intervention and community-based research, were used (table 1).

**Table 1:** A sample of the search string was used in the study

Databases (hits)	Key words used
PubMed (n=467) Science Direct, Google scholar and Cochrane Library (n=1643)	(1) physical activity; (2) physical inactivity; (3) exertion; (4) fitness; (5) community-based intervention; (6) community-based research; (7) population-based intervention; (8) community-based research; (9) 1 or 2 or 3 or 4 or 5 or 6 or 7 or 8; (10) randomised controlled trial; (11) controlled trial; (12) 9 and 10; (13) 9 and 11 Limit 12 and 13 to all women (18–65 years old) and English and humans

First, duplicate articles were removed by using End Note Software and then any remaining duplicate articles were deleted manually.

We used an iterative approach, which maximises the specifications of the search scope, to find the key literature. Additional web searches were performed after extracting relevant information, such as key words, phrases and authors, from the articles within the field of PA and community-based research (snowball search). The title and abstract of all potentially relevant articles were screened by two reviewers (LAF and OR) in order to find applicable information about PA promotion in the community-intervention section. If the abstract did not have sufficient information, the full text of the article was screened for further information. Any discrepancies between the two reviewers were resolved with discussions and consensus. If the reviewers could not reach a final conclusion, the article was investigated by the third reviewer (MA-L). The inclusion and exclusion criteria for selecting the studies were shown on the basis of PICOS in table 2.

**Table 2:** The inclusion and exclusion criteria for selecting the studies on the basis of PICOS

PICOS criteria	
Participants	Participants were to be 18–65 years of age. The study did not involve disease-state populations (for example multiple sclerosis rehabilitation patients.
Interventions	Interventions must be designed to improve PA and to prevent physical inactivity, cardiovascular disease, diabetes and other side effects of sedentary life style. The study only included community-based interventions.
Comparisons	Studies must provide an assessment of an intervention group through comparison with a control or comparison group which was simultaneously derived from the same or similar settings.
Outcomes	Participants were to be 18–65 years of age. Participation in PA must be one of the measured outcomes. Studies must at least demonstrate a specific measure of PA (objective, self-reported or both) at the baseline and follow-up.
Study design	In this review articles with both random and non-random allocation of participants to study groups were included, but results from observational studies were not reported.

### Assessment of methodological quality

Quality assessments of studies were performed using the information available in the articles through the critical appraisal sheet. This appraisal is composed of seven scales including Delphi List, PEDro, Maastricht, Maastricht-Amsterdam List, Bizzini, vanTulder and Jadad. The appraisal was compiled in a set of 39 items by Olivo and *et al*,^24^ where the items were divided into five categories: patient selection, blinding, interventions, outcomes and statistics (table 3).

**Table 3:** Methodological quality scores and ratings

Study	Item scoring																																							
	Patient Selection						Blinding						Interventions															Outcomes						Statistics						Scores/ Rating
	1	2	3	4	5	6	7	8	9	10	11	12	13	14	15	16	17	18	19	20	21	22	23	24	25	26	27	28	29	30	31	32	33	34	35	36	37	38	39	
Albright *et al*^28^	1	1	0	0	0	1	NA	NA	0	NA	NA	0	1	0	NA	NA	NA	1	0	1	0	1	0	1	0	1	1	1	1	1	1	0	1	1	1	0	0	1	NA	0.56/high
Gaston *et al*^32^	1	0	0	0	0	1	NA	NA	1	NA	NA	0	1	0	NA	NA	NA	0	0	1	0	0	0	1	1	1	1	1	1	0	1	0	1	1	0	0	0	0	NA	0.44/low
Keyserling *et al*^21^	1	1	1	1	1	1	NA	NA	1	NA	NA	0	1	0	NA	NA	NA	0	0	1	1	1	0	1	1	1	1	1	1	1	0	1	1	1	1	1	1	1	NA	0.78/high
Lombard *et al*^29^	*1*	1	1	1	1	1	NA	NA	1	NA	NA	1	1	0	NA	NA	NA	0	0	0	0	0	0	1	0	1	1	1	1	0	0	1	1	1	1	1	0	1	NA	0.62/high
Napolitano *et al*^30^	1	1	0	0	0	1	NA	NA	1	NA	NA	0	1	1	NA	NA	NA	0	0	1	1	0	0	1	1	1	0	1	1	1	1	0	1	1	1	0	0	1	NA	0.59/high
Pazoki *et al*^35^	1	1	0	0	0	1	NA	NA	0	NA	NA	0	0	1	NA	NA	NA	0	0	0	0	0	0	1	0	1	1	1	1	0	0	0	1	1	0	0	1	1	NA	0.4/low
Ransdell *et al*^33^	1	1	0	0	0	0	NA	NA	0	NA	NA	0	1	0	NA	NA	NA	0	0	1	0	0	0	1	0	1	1	1	1	1	1	1	1	1	1	0	0	1	NA	0.5/low
Sharpe *et al*^34^	1	0	0	0	0	1	NA	NA	0	NA	NA	0	1	1	NA	NA	NA	0	0	1	0	0	0	1	0	1	1	1	1	0	0	1	1	1	1	0	0	1	NA	0.47/low
Yancey *et al*^31^	1	1	1	1	1	1	NA	NA	1	NA	NA	0	1	1	NA	NA	NA	0	0	0	0	1	0	1	1	1	1	1	1	1	0	1	1	1	1	1	0	1	NA	0.72/high

1, eligibility criteria; 2, described as randomised; 3, randomisation performed; 4, randomisation described as appropriate; 5, randomisation concealed; 6, baseline comparability; 7, described as double blind; 8, blinding described as appropriate; 9, blinding of investigator/assessor;10, blinding of subject/patient; 11, blinding of therapist; 12, blinding of the outcome (results); 13, treatment protocol adequately described for the treatment and control groups; 14, control and placebo adequate; 15, cointerventions avoided or comparable; 16, cointerventions reported for each group separately; 17, control for cointerventions in design; 18, testing of subject adherence; 19, adherence acceptable in all groups; 20, description of withdrawals and dropouts; 21, withdrawals/dropouts rate described and acceptable; 22, reasons for dropouts; 23, adverse effects described; 24, follow-up details reported; 25, follow-up period adequate; 26, short follow-up performed; 27, timing of outcomes comparable in all groups; 28, description of outcome measures; 29, relevant outcomes included; 30, validity reported for main outcome measure; 31, reliability reported for main outcome measure; 32, responsiveness reported for main outcome measure; 33, use of quantitative outcome measures; 34, descriptive measures reported for the main outcome; 35, appropriate statistical analysis included; 36, sample size calculated a priori; 37, adequate sample size; 38, sample size described for each group; 3, intention-to-treat analysis included; NA, not applicable.

Each item listed in the critical appraisal sheet was specified by the score of one if it was included in the article, and specified by the score of zero if it was not included in the article or if the information provided by the authors was not sufficient to make a clear statement. In the case where a study did not consider a particular item, the item was marked as inapplicable in the critical appraisal sheet. The total score of each study was calculated by dividing the number of items included by the number of applicable items. The range of scores fell between zero and one. Finally, studies were graded based on the number of items that they had in the critical appraisal sheet.^24^ If the score was between 0 and 0.5, it was considered a low methodological quality study, and if the score was between 0.51 and 1, it was considered a high methodological quality study.

The critical appraisal was independently completed by the two reviewers (LAF and OR), and the results were compared. Disagreements between the two reviewers were discussed during a meeting to achieve consensus. If they could not reach an agreement, the third reviewer (MA-L) was consulted to make the final decision.

### Data extraction

Standardised data extraction forms were prepared through consultation with a methodological expert. They were then verified and completed by one reviewer (LAF), and furthermore checked by another reviewer (MA-L) for accuracy. The extracted data included the title, year, country, design of the study along with participant characteristics, randomisation procedure, intervention description, control or comparison groups, length of follow-up, measure of PA, health indicators and main results.

During the data extraction process additional details were considered: was it a theory-based intervention?; which constructs did the researchers use?; what was the number of participants at the baseline, the end of intervention and follow-up?; what was the effectiveness of the main outcome measures for assessing the level of evidence?; and, what were the details regarding the specific intervention and type of measurement tools (objective or subjective tools)?

### Strength of evidence and data synthesis

Heterogeneity in the type of interventions prevented reviewers from conducting a meta-analysis of the studies; therefore, narrative synthesis was used. As previously used in best evidence syntheses, conclusions regarding the effectiveness of programmes on PA outcome measures were drawn using a rating system referencing the levels of evidence on the basis of study design and methodological quality.^25–27^ ¸Five levels of evidence were defined: (1) strong evidence: at least 2 RCTs of high quality with ‘consistent’ (significant) results; (2) moderate evidence: 1 RCT of high quality and at least 1 RCT of low quality, or 1 RCT of high quality and at least 1 controlled trial of high quality (for both situations, consistent results were required); (3) limited evidence: 1 RCT of high quality and at least 1 controlled trial of low quality or more than 1 RCT of low quality or more than 1 controlled trial of high quality (for all situations, consistent results were required); (4) inconclusive evidence: only 1 study or multiple-controlled trials of low quality or contradictory results; and (5) no evidence: more than 1 study with no significant or relevant results to a specific intervention. When the results of the studies were considered with regard to statistical significance, the p value was less than 0.05. If at least 75% of each of the relevant studies were reported to have significant results in the same direction then we considered the overall results to be consistent'. In a stratified analysis we assessed and reported levels of evidence for studies according to type of intervention.

**Table 4:** Characteristics of included studies

Study (year)^ref^	Country	Design of study	Population (n)	General intervention	Outcome measure	Measurement times	Results
Albright *et al* (2005)^28^	USA	CT	Baseline: Phone+Mail Counselling group=35, Mail Support group=37; there are no exact information for follow-ups	All participants received 2 months of Weekly 1 h classes (group activities, small-group discussions, and question-and-answer games). Random allocation to 10 months of either home-based telephone counselling, additional information, and feedback for PA by mailed newsletters (Phone+Mail Counselling condition) or only the mailed newsletters (Mail Support condition).	PA	Baseline, 10 weeks, 6 and 12 months after baseline	There was not any significant difference in the percentage of participations in 30 or more of MVPA at least 5 days weekly between the Phone+Mail Counselling group (49%) and Mail Support group (35%) after 12 months Results of between group comparison after 10 months showed, there is a significantly greater increase in total energy expenditure via PA in phone+mail counselling group than the mail support group (p<0.05)
Gaston *et al* (2007)^32^	USA	CT	Baseline: intervention group=106, comparison group=28; follow-up (10 weeks): intervention group=83, comparison group=23; follow-up (6 months): intervention group=?, comparison group=?, sample size in both groups: 42; follow-up (12 months): intervention group=45, comparison group=7	Intervention group received curriculum-, culture- and gender-specific based approaches to behavioural change. Participants were divided into 10 structured intervention groups with 8–13 women per group. The groups met for 90 min for 10 weeks, led by facilitators and received the Gaston book. All participants signed a group contract for improving PA at the first session. Control group received the copy of the Gaston but did not receive a curriculum, facilitator and expert consultants.	Perception of overall health, Health attitudes, eating patterns and PA	Baseline,10 weeks, 6 and 12 months	Statistically significant increase in the women’s involvement in aerobic exercise (from 1.9 day per wk at baseline to 3.97, 2.48 and 3.21 day per week at 10 weeks, 6 and 12 months). A significant.10-week difference was found in the women’s diet, with them reporting eating more nutritious foods (p<0.001)
Keyserling *et al* (2008)^21^	USA	RCT	Baseline: EI group=118, MI group=118; follow-up (6 months): EI group=108, MI group=110; follow-up (12 months): EI group=106, MI group intervention=106	EI group received two 6-month phases, first phase included 3 group sessions, 2 individual counselling sessions, and 3 phone calls from an expert counsellor; second phase (maintenance phase) included 1 individual counselling session and 7 monthly peer counsellor calls. MI group consisted of a one-time mailing of pamphlets on PA and diet	PA, Dietary intake, fasting blood lipids, blood pressure, weight, and psychosocial variables	Baseline, 6 and 12 months	PA outcomes by using a questionnaire: at the 6 and 12 month follow-up, the EI group reported significantly more moderate (p=0.001) and vigorous (p=0.03) PA and there is no significant differences between EI and MI groups by using accelerometer at 6 and 12 months(p>0.05). Dietary intake improved more in the EI compared to the MI (questionnaire at 6 and 12 months, p=0.001; serum carotenoid index, p=0.05)
Lombard *et al* (2009)^29^	Australia	RCT	Baseline: intervention group=127, comparison group=123; follow-up (4 months): intervention group=88, comparison group=85	Intervention group consisted of 4 sessions including 3 1 h interactive group sessions in the first month plus one review session at 4 months; Content included messages with clear goals on PA and behaviour change. Participants were encouraged to enter voluntary school-based walking groups or to walk with friends for social support; moreover, they used pedometer for self-monitoring, and received one instruction for using it. Comparison group participated in individual 30 mins, non-interactive health education group lecture and received a pedometer without setting the goal.	PA, diet, self-monitoring, self-efficacy, anthropometric measures	Baseline and 4 months	The intervention group reported more improvements in PA level even though the difference between the two groups was not significant. The difference in scores between groups on the basis of MET was 35 (−315 to 416), 67 (−389 to 525), 46 (−412 to 506) for walking, moderate and vigorous PA, respectively Participants in both groups decreased weight with no significant difference between groups (p=0.95)
Napolitano *et al* (2006)^30^	USA	CT	Baseline: CTM=93, Jumpstart=95, Wellness control=92; number of participants did not report for follow-ups 1, 3, 6 and 12 months	CTM group received a 12-week programme and one mailing booklet covering a topic weekly on the basis of American Heart Association guideline for PA. The Jumpstart group completed a 65-item questionnaire for assessing the stage of change, processes of change and self-efficacy related to PA at baseline and prior to the 1-month, 3-month and 6-month time points. Participants received a booklet matched to stage of change and an individually tailored feedback report addressing barriers, benefits, self-efficacy, social support and goal setting. The Wellness group received one mailing of women’s health information about sleep, cancer prevention, and nutrition.	PA, stage of change, process of change, self-efficacy	Baseline, 3 and 12 months	At 3 months, participants in the Jumpstart group reported significantly more minutes of PA per week than participants in the Wellness group (respectively, 140.4±14.82, 98.1±15.09) (p<0.05). The Jumpstart group showed an inclination towards significance (p=0.054) when compared with the CTM group (99.5±15.11); there was no significant difference between the CTM and Wellness groups. At 12 months, no significant differences existed between any of the treatment groups
Pazoki *et al* (2007)^35^	Iran	CT	Baseline: Intervention=179, control=179; follow-up (8 weeks): Intervention=170, control=160	The intervention group received an 8-week programme related to the American Heart Association PA for women, audio-taped activity instructions and an educational package which were given weekly thorough home-visits by local volunteers. Control group: not reported.	PA	At baseline and 8 weeks	Increasing in PA from 3% and 2.7% at baseline to 13.4% and 3% after 8 weeks in the intervention and control groups. Intervention group reported more minutes of PA per wk(mean=139.81, SE=23.35) than women in the control group (mean=40.14, SE=12.65) at week 8 and the difference between two groups was statistically significant (p<0.0001)
Ransdell *et al* (2003)^33^	USA	CT	Baseline: home-based intervention=20, community-based intervention=20; follow-up: home-based intervention=14, community-based intervention=20	CB activities were completed at a fitness facility within a university and met 3 times per week and fitness-oriented activities 2 times a week, Fitness activity days lasted 60–75 min and consisted of a 5 min warm-up, 20–30 min of aerobic activity, 20–30 min of weight training, and 5–10 min of stretching and abdominal strengthening exercises. HB group received a detailed packet including a calendar of recommended activities, pictures of various stretches, and strength-training activities, plus some advice for overcoming barriers, and sent or faxed their PA logs to the lead author every 2 weeks.	PA, health-related fitness, BP	At baseline and 12 weeks	There were no differences between changes in PA for home-based and community-based groups. Mothers and daughters in both groups significantly increased their participation in aerobic, muscular strength, and flexibility activities (p=0.02 to 0.000).
Sharpe *et al* (2010)^34^	USA	CT	Baseline: full intervention=430, community-media exposure only=245, no intervention: 234 ; follow-up: full intervention=217, community-media exposure only=820, no intervention: 822	Intervention consisted of two components: (a) a year-long media campaign to increase moderate-intensity exercise, where 27 women from the community were social models and their photos were in print and television sports and participated in the 13 monthly exercise events. (b) Behavioural intervention consisted of an intensive, 24-week, minimal-contact intervention which included an orientation packet and weekly tips, a pedometer, a guide to exercise and a goal-setting. Women participated in group exercise which gave them an opportunity to enjoy new activities and meet exercise partners. Group 1 received the full intervention, enrolled in the full 24-week behavioural programme and was exposed to countywide media messages. Group 2 lived in the media exposed county but not enrolled in the behavioural intervention. Group 3 received no intervention.	PA, self-efficacy, anthropometric in formation, counting steps	Baseline and 12 months	Behavioural intervention with media messages was more effective than media messages alone or no intervention Women in the behavioural intervention had the greatest pre- to post-programme positive differences and had significantly higher recall of programme ads and of the main message than did the media only sample
Yancey *et al* (2006)^31^	USA	RCT	Baseline: intervention=193, ccontrol=183; follow-up (2 months): intervention=158, control=156; follow-up (6 months): intervention=118, control=110; intervention (12 months)=135, control=128	The intervention group included 8 weekly 2 h interactive group sessions, skills training in a regular exercise regimen, interview by a dietician about their food intake 3 or 4 times during the intervention and feedback on the quality and adequacy of their intake. Participants were encouraged to invite one close female relative or friend to accompany them during postintervention use of health club facilities. Control group received 8 weekly, 2 h interactive group sessions on health topics such as tobacco and cancer screening without the external social support.	BIA, WHR,BMI, sedentary behaviour, PA, fitness	At baseline, 2, 6, and 12 months	There was a significant effect of the intervention on PA at 2 months (p=0.0148), remaining marginally significant at 12 months (p=0.058) Participants in the intervention group showed a trend toward weight stability at 2 months compared with control group (p=0.75; p=0.08, respectively), disappearing at 12 months (p=0.0001; p=0.001, respectively)

BIA, bioelectrical impedance analyser; CAB, Community Advisory Board; COMBO, pedometer-plus group; CTM, choose to move; EI, enhanced intervention; MET, metabolic equivalent; MI, minimum intervention; MVPA, moderate-to-vigorous physical activity; PED, pedometer-only group; TTM, trans-theoretical model; WHR, waist/hip ratio.

## Results

**Figure 1:**
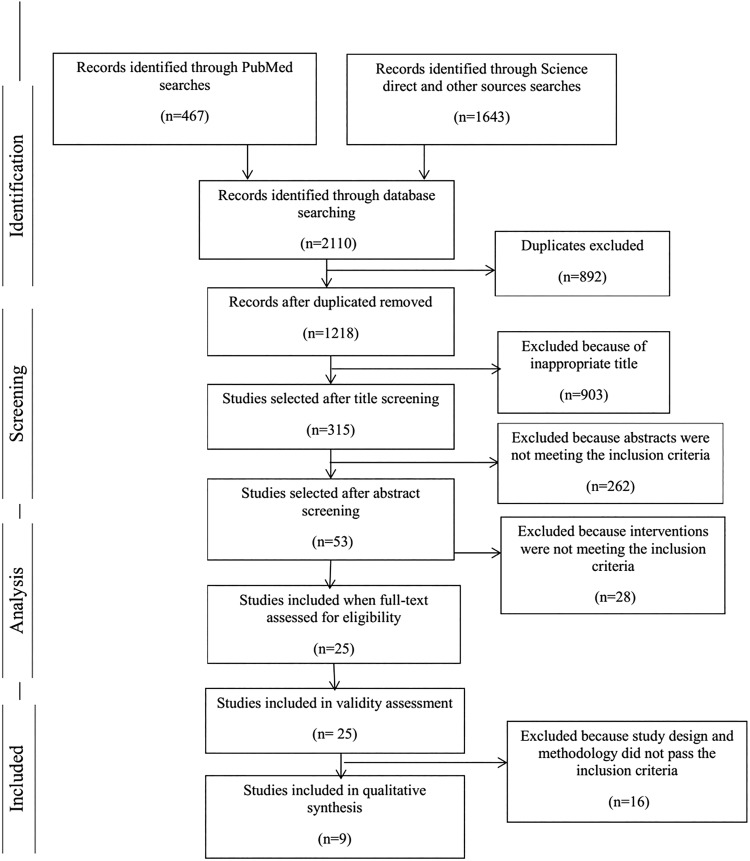
Flow diagram used for the identification, screening, eligibility and inclusion of studies.^23^

Overall, the initial search identified 2110 publications. After deduplication, 1218 relevant articles remained. At first the screening of titles led to 315 potentially relevant articles. The abstract content of all 315 studies were then screened and finally 53 articles remained, which had inclusion and exclusion criteria of systematic review on the basis of titles and abstracts assessment. The full text of the 25 studies were included for details assessment, resulting in 16 articles being excluded (figure 1). The reasons for exclusion were irrelevant outcomes for this review (n=5), PA interventions without evaluation (n=4), non-community-based intervention (n=2), involvement of disease-state populations and participants who were more than 65 years of age in the study (n=3), publication of two similar articles in different journals (n=1) and the use convenience sampling (n=1).

Nine articles were selected from this literature review. Table 4 provides the characteristics (ie, population, general intervention, outcome measure, measurement times and results) of all studies included in the review.

### Methodological quality

Table 3 shows the methodological quality of the included studies. Agreement was 92.6% on the 325 items scored through the quality assessment. Full consensus on all items was reached after discussion between the two reviewers. Five of the 9 articles were considered high quality.^21^ ^28–30^ ^31^ There was not sufficient information about random allocation used in most studies as only 3 of these (33%) described random allocation^21^ ^29^ ^31^ and only 3 (33%) provided sufficient information about allocation concealment at the time of outcome assessment.^21^ ^29^ ^31^ There were blinding issues due to nature of PA interventions as it was not possible to blind participants to the types of intervention. However, some studies used blinding of investigator/assessor and statistician to increase study accuracy. Five studies (55%) applied blinding of the investigator^21^ ^29–32^ and 1 study (11%) solely used blinding of statistician.^29^ Most studies had similar periods which passed before conducting the outcome assessment. Only 4 studies (44%) had a follow-up of 3 months or longer.^21^ ^30–32^

### Study characteristics

Seven of 9 studies were carried out in the USA,^21^ ^28^ ^30–34^ 1 in Australia,^29^ and 1 in Iran.^35^

The intervention studies were categorised as: physical activity only, nutritional and physical activity interventions. There were 5 of nine articles where programmes were designed to modify PA^28^ ^30^ ^33–35^ and the remainder were designed as both PA and nutritional interventions.^21^ ^29^ ^31–32^

The most common duration for interventions was 12 months.^21^ ^28^ ^34^ Other interventions lasted 8 weeks,^31^ ^35^ 10 weeks^32^ or 4 months.^29^

All of the studies were designed on the basis of a multicomponent approach. All studies evaluated social science theory-based interventions; seven of nine studies used applied social cognitive theories,^21^ ^28–30^ ^32^ ^33^ ^35^ while 1 used both SCT and social marketing theory (SMT),^34^ and another used the social ecological model.^31^ The most common constructs of SCT were used, including social support, goal setting, overcoming potential barriers and self-monitoring. Some studies have emphasised specific constructs or applied particular interventions that did not exist in other studies. For example, Albright *et al*^28^ used verbal encouragement and written reinforcement to achieve short-term and long-term PA goals. Gaston *et al*^32^ and Pazoki *et al*^35^ used cultural facilitators and expert consultants for teaching behavioural strategies and skills to help the women implement an individualised health plan. Keyserling *et al*^21^ gave contact information to participants for local healthy PA resources. Lombard *et al*^29^ offered problem-solving training for overcoming the barriers of PA. Ransdell *et al*^33^ used a daughter and mother exercise strategy to produce social support and motivation to increase PA. Sharpe *et al* used media messages for promotion of PA.^34^ Yancey *et al*^31^ applied an economic incentive of a free 1-year gym membership for all participants.

Measurement of PA was mostly focused on self-report questionnaires or recall instruments (using different types of PA questionnaire). Four of nine articles used both self-report questionnaires or recall instruments and pedometers for measurement of PA.^21^ ^28–29^ ^34^

### Evidence of effect on physical activity

Seven studies reported a positive intervention effect (77.7%), and in 4 of these studies statistical significance was achieved (44.45%). Significant results ranged from an increase of 2.07 days per week in doing aerobic exercise to a 10.4% increase in participation in regular PA (at least 30 min of moderate intensity PA for at least 5 days a week, or at least 20 min of vigorous PA for at least 3 days a week).

Seven studies evaluated social cognitive theory-based interventions, including 2 high-quality randomised controlled trials,^21^ ^29^ 2 high quality controlled trials^28^ ^30^ and 3 low quality controlled trial.^32^ ^33^ ^35^ Two of these studies were high quality and randomised controlled trials,^21^ ^29^ but had no statistically significant intervention effect; therefore, there was no evidence on the basis of effectiveness for social cognitive theory-based interventions.

With regard to other social science theory-based interventions, there was only 1 low quality controlled trial intervention accomplished on the basis of a mix of SCT and SMT, and 1 high-quality randomised controlled trial which used the social ecological model.^31^ ^34^ These two articles illustrated the inconclusive evidence of intervention effectiveness.

## Discussion

### Summary of evidence

The purpose of this systematic literature review was to assess the effectiveness of community-based PA interventions for women. Many studies were found in the literature, but a very small number of studies were community-based interventions performed among women or met the inclusion criteria of this study. Consequently, this problem brought about a small number of studies being included in the review. Most of these studies modified PA and were multicomponent interventions. However, reviewers attempted to categorise the studies in a meaningful and logical model, but were unable to recognise any consistent evidence to support the effectiveness of community-based interventions to enhance PA level. Heterogeneity existed between the types of interventions, intensity of activities, study designs, the duration of follow-ups and assessment tools. Reviewers found that social cognitive theory-based interventions had no evidence of an effect of interventions on PA and the evidence of an effect for other social science theory-based interventions was inconclusive. Most of these studies were not random and did not have any statistical significance. More high quality and randomised studies are required to strengthen and confirm these results. In overall, due to specific characteristics of interventions, reviewers could not determine which type of interventions, intensity, frequency or type of PA were successful in promoting PA among women.

### Implementation of interventions

Results showed that most of the articles were limited or had inconclusive evidence of an interventions’ effect. There were many factors which contributed to the restricted effectiveness of interventions: small sample size, small power to detect differences between groups, baseline differences between groups, the intensity levels of interventions, lack of wait-list control group by comparing the intervention group results with another intervention type or minimal intervention, and adherence. Several studies which were included had these problems. For example, all of the papers described did not have acceptable adherence and most of them did not have a control group. All studies had a sociological basis; however, even those that used same theories had different constructs.

### Limitations and recommendation for future studies

There are a number of limitations to this study. First, reviewers limited the search to English language articles and did not include other language interventions, such as German or Italian. Second, the search strategy covered resources published between 2000 and 2013 as the process of conducting the systematic review and reviewing the article was long.

Third, due to the small number of included papers and the lack of statistically significant differences, the results of this review are difficult to interpret. Fourth, methodological limitations across studies included the short time of intervention or follow-up, insufficient adjustment for potential confounders, lack of randomisation procedure and blinding at outcome assessment. Fifth, there was a lack of precision in the measurement of PA outcomes in some studies. Sixth, a conclusive meta-analysis cannot be achieved with these studies because of the heterogeneous nature of these studies and explanations cannot be made concerning the effect size of the interventions. Seventh, reviewers could not distinct biased publications that only reported positive findings in community-based interventions for PA improvement as these publications were some of the available resources. Reviewers also faced the challenge that measures of PA differed markedly and were reported both as indirect and direct measures. Though reviewers had planned a priori to conduct subgroup analysis of direct (eg, accelerometer or pedometer) versus indirect (eg, self-report) measures of PA, this was not possible because of the heterogeneity of measurement tools and interventions.

To have a fair assessment, future studies on PA measurement should have similar approaches and tools. There is a need for more rigorous research designs, including higher quality randomised controlled trials in this age group and culture-based multicomponent and community-based intervention programmes that consider either individual or environmental factors for changing PA levels

One of the goals of the community intervention is to design programmes that include the majority of the population, but it seems including personal desires and interests into the design of PA programmes could provide better results. One intervention approach may not fit all, therefore, different approaches should be offered: some people may prefer the private feedback from a device such as pedometer; others may respond to interventions delivered through the internet, others may benefit from the social support in doing a PA group, whereas others may increase PA in response to telephone counselling or facilitator counselling.

In community-based interventions, the number of participants that contribute in all levels of measurement, design, application and assessment increase the chance of success for an intervention programme. At the same time, the efficacy and reliability of an intervention programme is more important than the number of people that an intervention could involve.

## Conclusion

To our extensive search, this is the first published systematic review aimed at community-based PA intervention studies for 18–65 years-old women. This review found low-quality to high-quality evidence of how to improve PA, although due to the inadequate supply of information reviewers could not determine which specific type, intensity, frequency or amount of intervention could significantly improve PA, or which intervention is more effective and sustainable. In addition, more studies are needed to address these gaps in knowledge for PA improvement among women. Based on the published evidence to date, it is necessary to conduct a multilevel approach for promoting PA. Reviewers have recognised the necessity of collaborations among community members, policymakers, as well as governmental and non-governmental organisations in developing more effective PA interventions for women.
